# Predicting BCI Subject Performance Using Probabilistic Spatio-Temporal Filters

**DOI:** 10.1371/journal.pone.0087056

**Published:** 2014-02-14

**Authors:** Heung-Il Suk, Siamac Fazli, Jan Mehnert, Klaus-Robert Müller, Seong-Whan Lee

**Affiliations:** 1 Department of Brain and Cognitive Engineering, Korea University, Anam-dong, Seongbuk-ku, Seoul, Republic of Korea; 2 Machine Learning Group, TU Berlin, Berlin, Germany; University of Maryland, College Park, United States of America

## Abstract

Recently, spatio-temporal filtering to enhance decoding for Brain-Computer-Interfacing (BCI) has become increasingly popular. In this work, we discuss a novel, fully Bayesian–and thereby probabilistic–framework, called Bayesian Spatio-Spectral Filter Optimization (BSSFO) and apply it to a large data set of 80 non-invasive EEG-based BCI experiments. Across the full frequency range, the BSSFO framework allows to analyze which spatio-spectral parameters are common and which ones differ across the subject population. As expected, large variability of brain rhythms is observed between subjects. We have clustered subjects according to similarities in their corresponding spectral characteristics from the BSSFO model, which is found to reflect their BCI performances well. In BCI, a considerable percentage of subjects is unable to use a BCI for communication, due to their missing ability to modulate their brain rhythms–a phenomenon sometimes denoted as BCI-illiteracy or inability. Predicting individual subjects’ performance preceding the actual, time-consuming BCI-experiment enhances the usage of BCIs, *e.g.*, by detecting users with BCI inability. This work additionally contributes by using the novel BSSFO method to predict the BCI-performance using only 2 minutes and 3 channels of resting-state EEG data recorded before the actual BCI-experiment. Specifically, by grouping the individual frequency characteristics we have nicely classified them into the subject ‘prototypes’ (like *μ* - or *β* -rhythm type subjects) or users without ability to communicate with a BCI, and then by further building a linear regression model based on the grouping we could predict subjects' performance with the maximum correlation coefficient of 0.581 with the performance later seen in the actual BCI session.

## Introduction

Classical Brain Computer Interfaces (BCIs) were based on operant conditioning [1], [2], [3], [4], [5], *i.e.*, the subject had to adapt the modulation of his/her brain rhythms. In recent years with the advent of machine learning methods in BCI, both - the subject and the computer - adapt; this has resulted in a reduction of calibration times and increased information transfer rates [6], [7], [8], [9], [10], [11], [12]. Machine-learning can help accurately model the spatio-temporal characteristics of a subject’s brain rhythms to ensure optimal decoding of the user’s intentions during feedback. For Sensory Motor Rhythms (SMR), Common Spatial Pattern (CSP) [13] and its variants are most commonly used [14], [15], [16], [17], [18], [19].

Within the last decade the performance in non-invasive EEG-based BCI has reached high levels of accuracy (up to 90%) in classifying EEGs into one of the predefined labels, *e.g.*, left-hand vs. right-hand motor imagery, nevertheless around 20% of the subjects show an inability to communicate with a BCI – sometimes also called BCI-illiteracy/inability [20], [21], [22], [23]. The reasons for BCI-inability are still under debate, however, for SMR-controlled BCIs, strong rhythms during resting state are found highly predictive for a later good online BCI performance [20]. Nevertheless, which frequencies will be most discriminative, depends on the individual subject physiology. The most common modulated frequency band used by a SMR-controlled BCI is the *μ*-rhythm around 10 Hz; a second target frequency band is the *β*-band around 20 Hz. Although most subjects modulate one or both of these frequency bands, they always show specific peak-frequencies while subjects with BCI-inability typically do not show any task-related modulation in these bands [20]. It is important to note that so far, SMR-controlled BCIs have been trained on one or two frequency bands only, the detailed spectrum was not considered until recently, where the first fully Bayesian approach has been introduced to the field: Bayesian Spatio-Spectral Filter Optimization (BSSFO) [24]. BSSFO allows to introduce prior knowledge into spatio-temporal filter optimization. It extracts a subject-specific filter distribution that can be analysed to gain a better understanding of individual differences of BCI users.

In this contribution, we will show that BSSFO not only yields a significant increase in classification accuracy over 80 subjects when compared to other spatio-temporal filter algorithms. But BSSFO filters may further be clustered across subjects according to the patterns corresponding to the extracted filter characteristics. We then analyze the resulting grouping in order to gain a better physiological understanding why some subjects perform better than others and what the characteristics of subjects with BCI-inability could be.

Our analysis extends [20], since we find an increased predictivity when using the full spectral characteristics of resting-state EEG measurements prior to the BCI. We further study the dependency of the prediction quality on the number of channels included. It should be noted that our analysis aims to get additional physiological insight to the phenomenon of BCI-inability; other protocols that involve *e.g.*, co-adaptive BCI [25], [26], [12], [27] can indeed help enable illiterates to communicate with BCI.

## Methods

**Figure 1:**
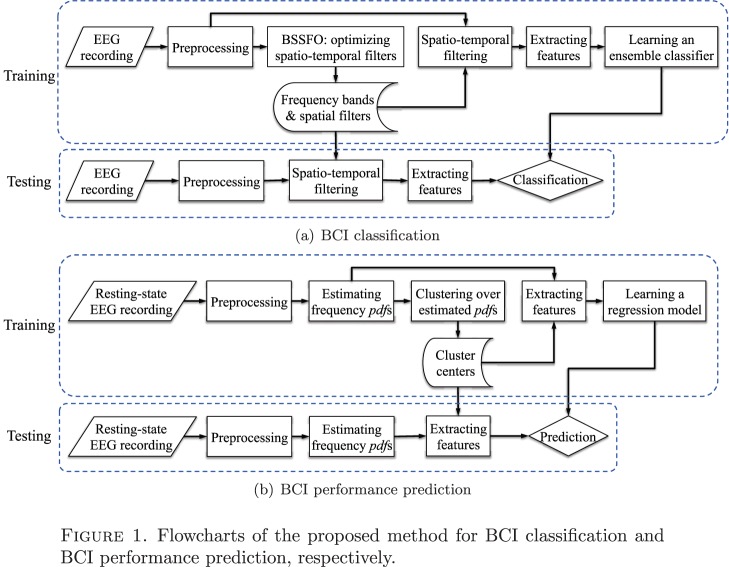
Flowcharts of the proposed method for BCI classification and BCI performance prediction, respectively.

In this section, we first describe the experimental data sets used to test the proposed fully Bayesian approach 1) for BCI classification and 2) for the prediction of subjects’ individual performance. We leave the mathematical background of the Bayesian framework to Appendix, in particular and how it constructs individual spatial and temporal filters used for the BCI classification. The proposed framework is compared to four competing methods. Furthermore, clustering of the derived patterns allows a physiological interpretation of the results. For the second aim of the study, *i.e.*, the prediction of the subjects’ performance, we formulate an application of the Bayesian framework for resting-state EEG data. In combination with a clustering of the derived spatio-temporal patterns, it enables us to analyze the predictability of these patterns for the subjects’ performance during the actual BCI and a physiological interpretation. In Fig. 1, we present flowcharts that outline the steps in data processing for each study, respectively. The detailed explanation for the steps is described below.

### 2.1 EEG Acquisition and Preprocessing

The EEG data used to evaluate the BSSFO algorithm has been acquired during a SMR-controlled BCI in a previous study [23], where 83 subjects performed motor imagery of three classes: Left-hand motor imagery (L), Right-hand motor imagery (R), and Foot motor imagery (F) to control a BCI. (The study was approved by the Ethical Review Board of the Medical Faculty, University of Tübingen. Each subject gave a written informed consent after having been informed about the purpose of the study.) Due to technical problems during the acquisition, 3 participants were excluded from the analysis. Subjects were seated in a comfortable chair and instructed to relax their arms, while these were lying on armrests. The recording was carried out with multichannel EEG amplifiers (BrainAmp DC by Brain Products, Munich, Germany) with 119 Ag/Ag/Cl electrodes and a nasion reference, and sampled at 1000 Hz with a band-pass of 0.05 Hz to 200 Hz. Vertical as well as horizontal ElectroOculoGram (EOG) and ElectroMyoGram (EMG) at both forearms and right leg were recorded, to ensure absence of artifacts within the EEG.

**Figure 2:**
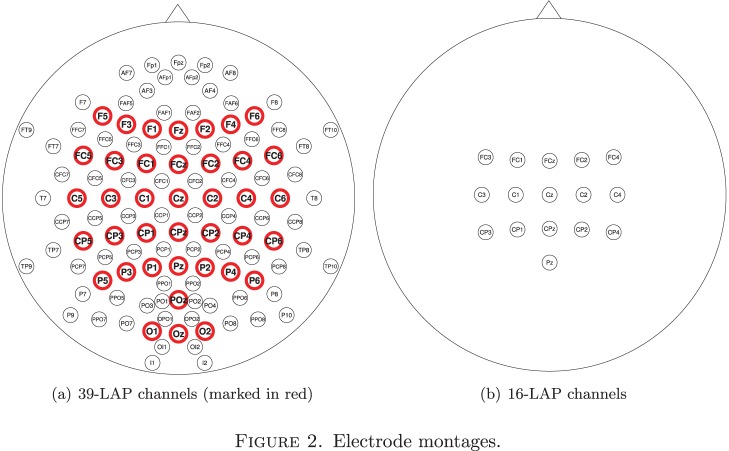
Electrode montages.

To test BFSSO for BCI in an off-line analysis (as described in Section 2.2), we only used data from motor imagery of the left and right hand recorded during three calibration sessions acquired during the described experiment, each consisting of 25 trials per class per subject, resulting in a total of 75 trials per class per subject. A single trial lasted for 8 seconds. At the beginning of each trial a crosshair appeared at the center of the screen for two seconds. After this initial 2 seconds, one of three possible visual cues in the form of an arrow pointing to the left, right, or downwards showed up for 4 seconds in a randomized order. The visual cues indicated the type of movement imagination to be performed by the participant. After the arrow disappeared, the screen was left blank for 2 seconds and then a new trial began. After every 20 trials a short 15-second break was given. The EEG data was downsampled to 100 Hz with a digital Chebyshev low-pass filter. Two sets of channels were defined and used for further analysis: a set of 39 LAPlacian (LAP)-filtered channels (Fig. 2(a)) and a second set of 16 LAP-filtered motor-related channels (Fig. 2(b)).

The study of Blankertz *et al.* further contained resting-state periods at the beginning of each of the 3 calibration sessions. In total, 10 periods of 15 seconds were recorded with the alternating tasks ‘relax with eyes open’ and ‘relax with eyes closed’. We pooled this resting-state data and used it to train BSSFO to predict the subjects’ BCI performance (see Section 2.3). We consider two channel arrangement schemes, namely, small channel arrangement (3-LAP: ‘C3’, ‘Cz’, ‘C4’.), large channel arrangement (16-LAP: the same channels used for the motor imagery experiment). For further details on the experimental and recording setup, please refer to [20].

### 2.2 Bayesian Spatio-spectral Filter Optimization for Decoding in Brain Computer Interfaces

**Figure 3:**
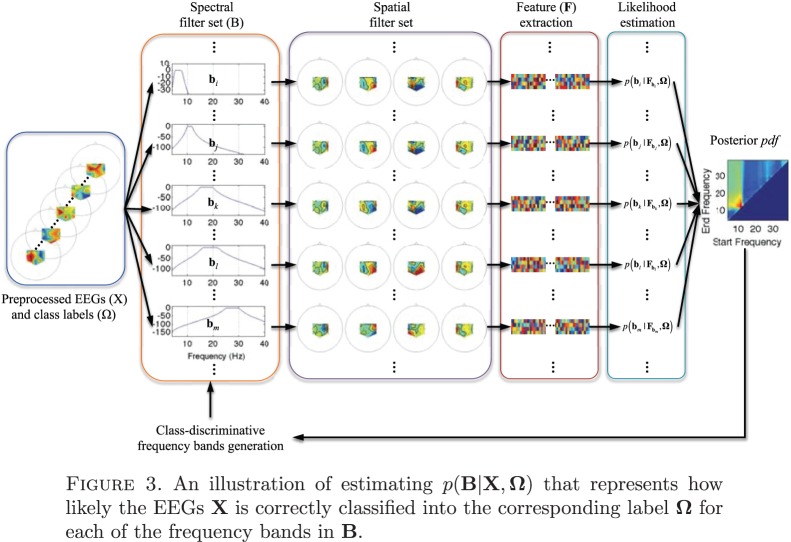
An illustration of estimating 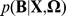 that represents how likely the EEGs X is correctly classified into the corresponding label Ω for each of the frequency bands in B.

**Figure 4:**
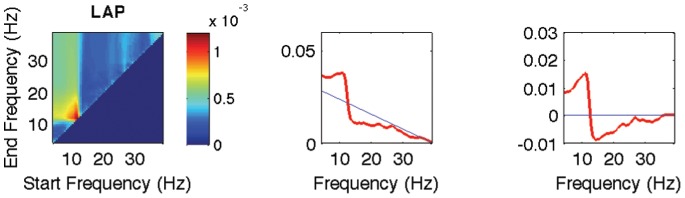
Estimation of a 1-dimensional *pdf* from a 2-dimensional *pdf* estimated by BSSFO. (Left) A 2-dimensional *pdf* estimated by BSSFO. (Middle) The red line represents the 1-dimensional *pdf* of the estimated 2-dimensional *pdf* and the blue straight line is the 1-dimensional *pdf* of a uniform 2-dimensional *pdf*. (Right) The red line is the uniform *pdf* subtracted version of the 1-dimensional *pdf*.

A schematic overview of the BSSFO method (see Appendix and [24]) is given in Fig. 3. Given a set of the preprocessed motor imagery EEG signals and a set of particles - each representing a specific frequency band sampled from a prior distribution - the BSSFO algorithm first filters the EEG signals for each frequency band. All the ensuing processes are based on information from this individual particle. The spectral filtering is followed by a spatial filtering, where a CSP is trained with the spectrally-filtered signals. The likelihood and the posterior *pdf* are then estimated on feature vectors extracted from the resulting filtered signals. This whole process is iterated until convergence or a predefined stopping criterion. We estimated class-discriminative *pdfs* for several frequency bands between 4 Hz and 40 Hz with an interval of 0.5 Hz. The result is then a 2 dimensional *pdf*, in which each dimension corresponds to a start and an end frequency, respectively (see *e.g.*, Fig. 4).

We applied the described process on experimental SMR-controlled BCI data and compared two channel configurations, already described in Section 2.1 to extract spatio-temporal filters for the BCI classification. The classification followed the same strategy as proposed by [24] except for the classifier. In the present study, we employed a Linear Discriminant Analysis (LDA) for classification. The resulting accuracies are compared with the the conventional CSP [16] on different band-pass filtering strategies. For the competing method, we also used a LDA as a classifier.

#### 2.2.1 Towards physiological interpretation of results from BSSFO

To enable a physiological understanding of the patterns resulting from BSSFO, we converted the estimated 2-dimensional *pdfs* into 1-dimensional *pdfs* as follows:
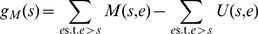
(1)where 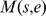 and 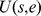 denote, respectively, the estimated 2-dimensional *pdf* and a uniform 2-dimensional *pdf*, and *s* and *e* are, respectively, the start and the end frequency for a band (see Fig. 4). The estimated 1-dimensional *pdf* is then weighted with a neurophysiological knowledge, represented by a mixture of Gaussians as 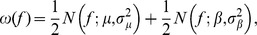 where *f* denotes a frequency. This final 1-dimensional *pdf* is used as input for a hierarchical clustering over all subjects. For each cluster, we derived a topographical map representing the average spatial patterns of the subjects belonging to the cluster. In addition to the clustering, we also computed the Pearson correlation between the Area Under the Curve (AUC) of the 1-dimensional *pdf* and the classification accuracy for each subject.

### 2.3 Prediction of BCI Performance from Resting-state Data

**Figure 5:**
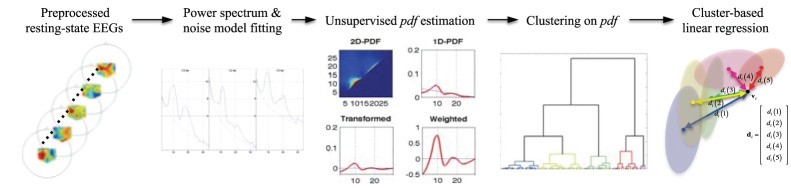
A schematic diagram of the BCI illiteracy prediction in a probabilistic framework.

The second aim of our study is to find spatio-temporal patterns in resting-state EEG data, which are predictive for the individuals BCI performance and, furthermore, allows to sort subjects along their frequency-type, *i.e.*, *μ* - and/or *β*-rhythm types, and ‘BCI-illiterates’. The basic principle follows the one described above: We first estimate a frequency *pdf* from a Power Spectral Density (PSD) of channels for each subject in an unsupervised manner (Section 2.3.1). A data clustering over the estimated *pdf* follows and finally we build a linear regression model using the cluster information (Section 2.3.2). A schematic diagram of building our BCI predictor is presented in Fig. 5.

#### 2.3.1 Unsupervised pdf estimation

In order to estimate a *pdf* of a frequency band, we first calculated the PSD in each channel individually with the preprocessed resting-state EEGs as follows:
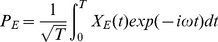
(2)where *t* denotes a time index, *E* denotes an electrode, and *X_E_* is a temporal EEG at the electrode *E*. For each PSD, the corresponding noise model is fitted as done in [20]:
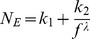
(3)where *f* denotes a frequency, and *k*_1_, *k*_2_, and *λ* are model parameters.

Based on those two ingredients, we extract the Frequency-Related Information (FRI) *S_E_* for each channel by taking the difference between a PSD and a noise model.
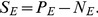
(4)


From an information theory point of view, Eq. (4) means that the smaller the value of *S_E_*, the less informative the frequency in the channel *E* is. This fact is utilized directly in our probability model described below.

Since the selection of a frequency band related to motor imagery tasks is one of the key issues in determining the classification performance, we build a *pdf* in terms of a frequency band. Following Suk *et al.’*s work [24], we represent a frequency band with a continuous random vector **B**. The problem is to estimate the *pdf* of this random variable 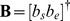, where *b_s_* and *b_e_* denote, respectively, the start and the end point of a frequency band, and † is a transpose operator.

We should note that given a set of preprocessed resting-state EEGs **X**, the posterior probability of a frequency band **B**, 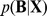, can be estimated indirectly from the set of FRIs 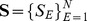, where *N* denotes the number of channels under consideration, as follows:
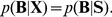
(5)


Taking into account channels, we can rewrite 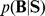 by a sum rule in a probability theory and a Bayes rule as follows:
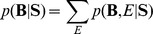





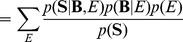





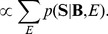
(6)With the application of a chain rule and the assumption of the uniform distributions for 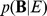 and 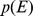, the last proportional relation can be derived.

From Eq. (6), all we need to do is to estimate the likelihood 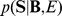. We define a likelihood for a frequency band **B** of the range *b_s_* and *b_e_* as follows:
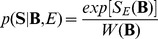
(7)where 
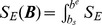
 and *W*(**B**) denotes a bandwidth.

At this moment, we should note that due to a computational issue, in this work, the PSDs and the corresponding noise models are computed and fitted every 0.5 Hz between 2 and 34 Hz, which covers both *μ* (8–12 Hz) and *β* (16–22 Hz) rhythms. Therefore, the domain for a start frequency is 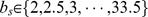 and that for an end frequency is 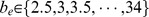.

**Figure 6:**
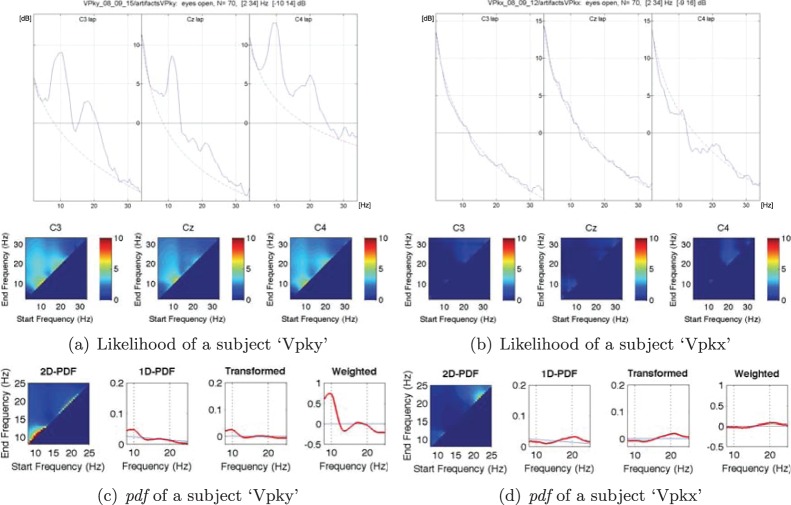
Estimation of the frequency-related resting-state EEG *pdf* with 3-LAP channels. In (c) and (d), the ‘Transformed’ *pdfs* are the uniform *pdf* subtracted version of the respective 1D-PDFs.

Let 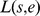 be the likelihood for the frequency band of *b_s_* and *b_e_* estimated from the resting-state EEGs, where *s* and *e* are, respectively, an index of the frequency value. The likelihood is computed by Eq. (7). Fig. 6(a) and Fig. 6(b) illustrate the examples of likelihood of three different electrodes for two different subjects. From the figures, we can see that a high power spectrum results in a high likelihood. From the likelihood we can naturally compute a *pdf* 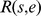 by normalization as follows:
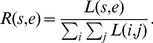
(8)


It is noteworthy that the probability represents the relative importance of a frequency band in a subject.

Although we can compute the probability of a frequency band from resting-state EEGs following the steps mentioned above, we cannot directly quantify the significance of the specific frequency band in terms of SMR-controlled BCI prediction, since the learning problem is now an *unsupervised* one. We, therefore, compute the likelihood for a noise model and contrast it to the likelihood from resting-state EEGs. In Eq. (7), 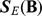 is defined as the difference between a PSD and the corresponding noise model. That is, the likelihood for a noise model becomes uniform 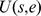 over all frequency bands. Then we can convert the 2D *pdf* into a 1D *pdf* 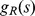 as done in motor imagery tasks:
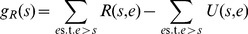
(9)where the indices *s* and *e* denote, respectively, a start and an end point of a frequency band.

However, once we convert a likelihood into a probability distribution, the original spectral power information disappears. Consequently, the probability distribution of different likelihoods can become similar between subjects even though their likelihoods are very different as exemplified in the leftmost matrix of Fig. 6(c) and Fig. 6(d). That is, while the likelihood of three electrodes for the two subjects are different from each other as shown in Fig. 6(a) and Fig. 6(b), after normalization of the probability density, the difference disappears. The probability represents the relative differences among values within a subject. Therefore, it is not meaningful to directly compare them between subjects for SMR-controlled BCI performance prediction. Therefore, to reflect the individual information of the PSD into the *pdf*, we multiply with a weight *η*, which we call a ‘subject-weight’, defined as the sum of the maximum power of each channel as follows:
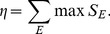
(10)


With the introduction of the subject-weight into the *pdf*, we can get a spectral power reflected density as shown at the rightmost matrix in Fig. 6(c) and Fig. 6(d). Note that after multiplication of the subject-weight, the resulting *pdf* does not meet the probability property anymore, *i.e.*, the sum of the values is not one. From the figures, we can clearly see the density differences between subjects while still keeping the relative significance of frequency within a subject. In addition, we also reflect the prior neurophysiological knowledge that *μ* - and *β* -rhythms are helpful for SMR-controlled BCI illiteracy prediction as proved in Blankertz *et al.’*s work [20]. Therefore, the *pdf* Ξ, which will be used for prediction, can be obtained as follows:
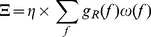
(11)where *η* denotes a subject-weight in Eq. (10) and 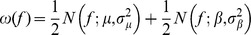 is a frequency-weight borrowed from the neurophysiological knowledge on motor imagery.

#### 2.3.2 Cluster-based linear regression

It is well-known that the spectral features with regard to the motor imagery are highly variable across subjects and a similar phenomenon can be observed in resting-state EEGs. We assume that if the spectral features of the resting-state among subjects are similar to each other, then their SMR-controlled BCI performance would be also similar. Therefore, we combine a clustering method with a linear regression method, but another possibility would be the use of a mixed effects model similar to [28]. For constructing the predictor, we first cluster the subjects based on their spectral feature vectors, and then learn a linear regression model based on the distance from the center of each cluster and the feature vectors. In this paper, we apply a hierarchical clustering method [29].

We utilize an augmented feature vector 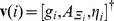, 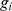 is a spectral *pdf* of the subject *i*, 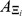 and 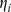 are, respectively, an AUC of 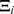 and a weight of the subject *i*. Due to the high dimension of the augmented vector and a small number of samples compared to the dimension, a principal component analysis technique is applied to reduce the dimension. We use the dimension-reduced feature vectors **v**(*i*) that include the information available from resting-state EEGs for clustering and the SMR-controlled BCI performance prediction.

In a hierarchical clustering, we use a ward criterion, which chooses the pair of clusters to merge at each step based on the optimal value of an objective function, *i.e.*, squared Euclidean distance:
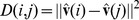
(12)where 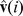 and 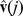 denote the dimension-reduced augmented feature vectors of the subject *i* and *j*, respectively. Since the hierarchical clustering method builds a hierarchy of clusters, it allows us to investigate the results from a physiological perspective.

**Figure 7:**
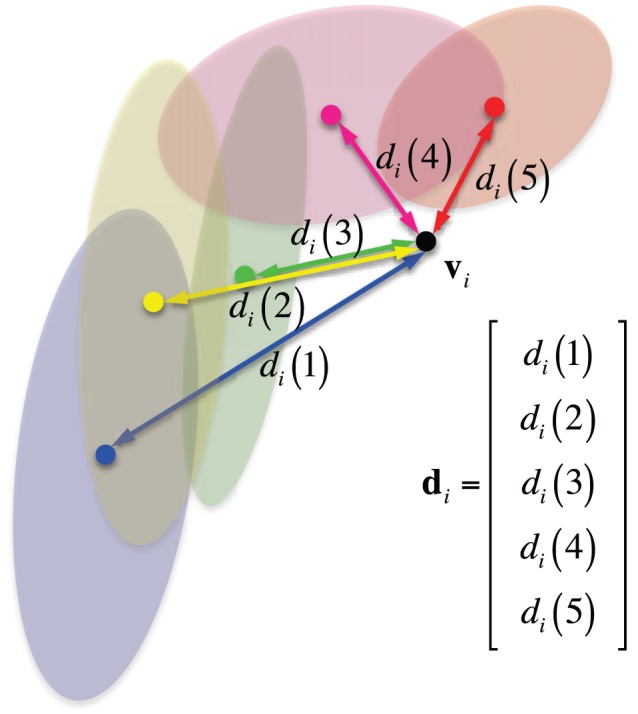
An example of constructing a cluster-distance vector for a subject *i* , which is the input to the linear regression function in BCI-performance prediction. The colored ovals represent a rough distribution of feature vectors labeled to the clusters and the dots represent the mean of each cluster.

In order for linear regression model fitting, we construct a new vector **d** *_i_* for each subject *i*, which consists of the distances from the center of clusters.
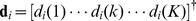
(13)where 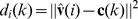, **c**(*k*) denotes the center of the cluster *k*, and *K* is the number of clusters. Fig. 7 illustrates the construction of a cluster distance vector. In the figure, each oval represents a rough distribution of the feature vectors 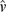 labeled to the respective cluster, and colors denote cluster labels. The dots in the center of each oval are the mean of the feature vectors assigned to the cluster.

With the cluster distance vectors 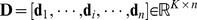, where *n* is the total number of subjects for training, we fit a linear regression model.
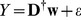
(14)where 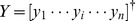 is a concatenated vector of the motor imagery accuracies over subjects, and *w* and *ε* are, respectively, a regression parameter and a bias.

Given a new subject’s EEG signal 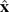, the SMR-controlled BCI performance for the subject can be predicted by.
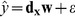
(15)where 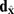 and 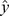 denote, respectively, a vector of distances between the dimension-reduced feature vector of the new subject and the center of clusters, and the predicted SMR-controlled BCI performance.

We used the same clustering method on the *pdfs* of the resting-state EEG data derived from Section 2.3 for a small and a large channel arrangement (see Section 2.1) to test whether also a small number of channels can lead to meaningful results. We then calculated a cluster-wise regression of the *pdf*s AUC and the subject’s performance in the later actual BCI session from Section 2.2, which gives insight whether belonging to a cluster can predict the BCI performance.

To gain further insight into the physiological features derived from the clustering, we calculated the first two principal components within the subjects belonging to each cluster. These principal components show the frequency pattern most common within a cluster. This was also done for small and large number of EEG channels to check whether a small number of channels still reveals meaningful results.

To find an appropriate number of clusters, we calculated the correlation between the resting-state predictor given by the clustering method and the actual BCI performance of the subject. We tested this for up to 20 clusters and for different channel arrangements.

Finally, we compared the clusters derived from the analysis of the actual BCI paradigm with the *pdf*s gained from the resting-state EEG data, in other words the discriminative and generative settings. As the clustering within the motor imagery sorted the subjects according to their performance, we are hereby able to show whether the resting-state *pdfs* show physiological meaningful predictor for the BCI performance.

## Results

### 3.1 Motor Imagery Classification and Physiological Interpretation of BSSFO Results

First, BSSFO is evaluated off-line for a large BCI data corpus of 80 subjects from [20]. (We performed 8-fold chronological cross-validation. In chronological cross-validation, since the time structure of the data is largely preserved, it can thus be considered as a relatively conservative measure. All parameters for temporal and spatial filters were estimated from training data in each of the cross-validation splits and applied to the test data. Regarding a loss function, 0–1 loss was applied.) BSSFO compares favorably to CSP with various strategies of band-power estimation (see Table 1). The band-pass filter strategies considered in this work were namely a broad-band filter (5–30 Hz), an *μ*-band filter (8–12 Hz), a *β*-band filter (16–22 Hz), and they were combined with CSP [16]. We also considered an established heuristic method for optimizing subject-dependent temporal filters [16]. Specifically, the log band-power of LAP-filtered EEG channels were computed from 5 to 35 Hz. Then the correlation coefficient of the band-power and the labels were calculated across all trials. We determine the frequency (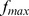) with the highest correlation coefficient. Based on this frequency, the band-pass frequency interval 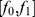 was increased, starting at 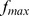 until 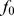 and 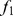 were smaller or equal to 5% of 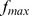.

**Table 1:** Comparison of the classification performance error among the competing methods for motor imagery.

Band [Hz]	16 Channels [%]	39 Channels [%]
Broad-band (5–3)	27.65±15.53	27.14±15.92
*μ*-band (8–12)	28.31±15.65	29.82±14.27
*β*-band (16–22)	39.09±12.51	38.45±12.56
Heuristic [12]	26.12±15.89	26.33±14.61
BSSFO [32]	**24.88±15.62**	**26.07**±**15.27**

**Figure 8:**
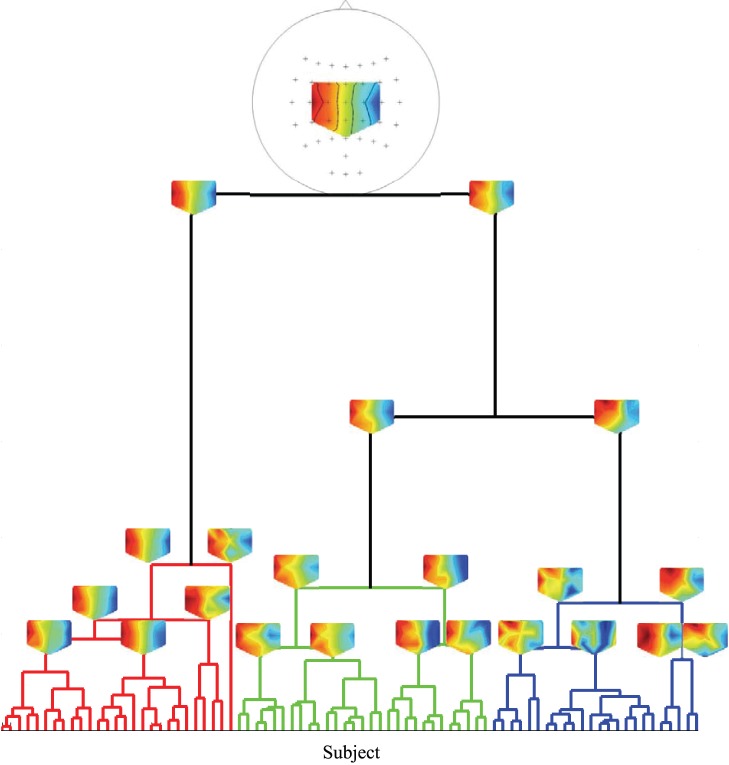
Results of the hierarchical clustering on 1D *pdfs* of the subjects on motor imagery tasks. The topographies present an average of the trained spatial patterns of the subjects belonging to the same cluster.

In order to gain a physiological interpretation of these encouraging results, a hierarchical clustering based on the 1 dimensional *pdf s* that are derived from the BSSFO’s 2 dimensional *pdf* of all subjects is computed. The resulting clustering into 3 groups is shown in Fig. 8 including an average of the 1D *pdfs* of the subjects belonging to one cluster shown as a topographical map. The first cluster (red) (Fig. 8, left hand side) has a very clear pattern with a strong lateralization between left- and right-hand motor imagery, which is also stable in the subgroups of this cluster. The pattern of the second cluster (green) (Fig. 8, middle) is less strongly lateralized and more occipital channels appear modulated only during right-hand imagery. They are contaminated by strong *α*-rhythms in the occipital cortex, which shares the frequency range of the *μ*-rhythm that we are actually interested in. Also subjects that belong to the second cluster show an overall smaller modulation than the one of the first cluster. The third cluster (blue) (Fig. 8, right hand side) exhibits considerable within-cluster-variance. This is already a first hint that a lower classification accuracy could be expected for the third group when compared to the others.

**Figure 9:**
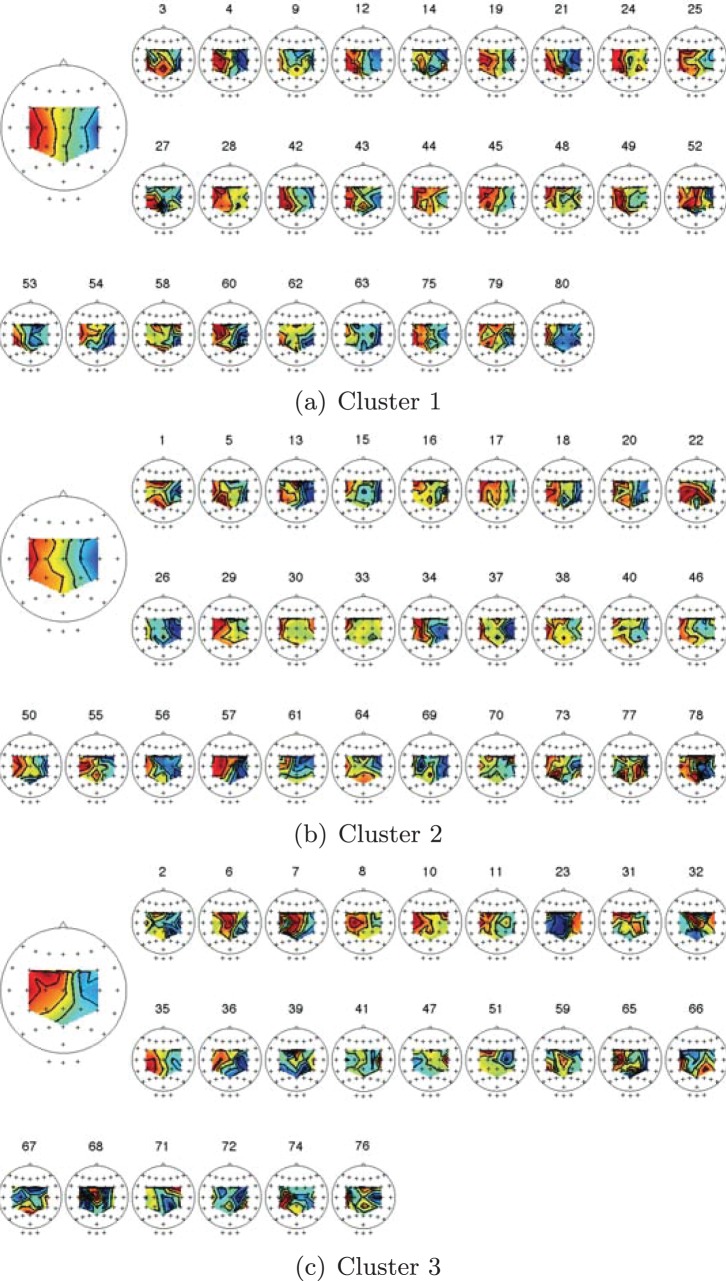
Illustration of the common spatial patterns of the subjects belonging to each cluster from Fig. 8. The results were obtained from the 16-LAP channel arrangement (see online color version of the figure).

To further investigate inter- and intra-cluster differences, we computed the mean spatial patterns of the 3 clusters. The results for each subject are shown in Fig. 9. Here, we gain a similar result as already mentioned above: Within the first cluster, we see a strong lateralization among nearly all subjects. This lateralization weakens in the second cluster and only exists among a few subjects of the third cluster.

**Figure 10:**
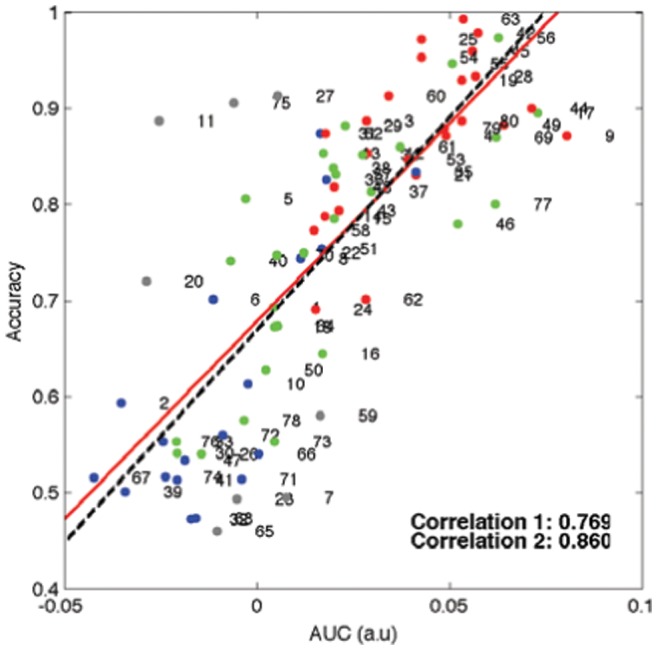
Pearson correlation of the frequency weighted area under curve (AUC) of 1D *pdf* with BCI performance. Each dot corresponds to a subject. The color of dots represents a cluster label in Fig. 8. The two lines represent a linear regression function for the values; the red solid line (correlation 1) is fitted to all the values considered and the black dotted line (correlation 2) is fitted for the values with outliers excluded.

We further computed Pearson’s correlation between the AUC of the 1 dimensional *pdf* and the classification accuracy. As shown in Fig. 10, the results are promising with a clear correlation between them: 0.769 with 16-LAP channels. If we remove outliers, the correlation increases to 0.860. From the figure, we can see that the clusters are also highly correlated with the accuracy. The subjects belonging to the red cluster mostly represent a high classification accuracy. Whereas, the subjects belonging to the blue cluster are distributed on the left-low corner of the graph indicating a low accuracy. The subjects in the green cluster are in the middle.

### 3.2 Prediction of BCI Performance with Resting-state EEG

**Figure 11:**
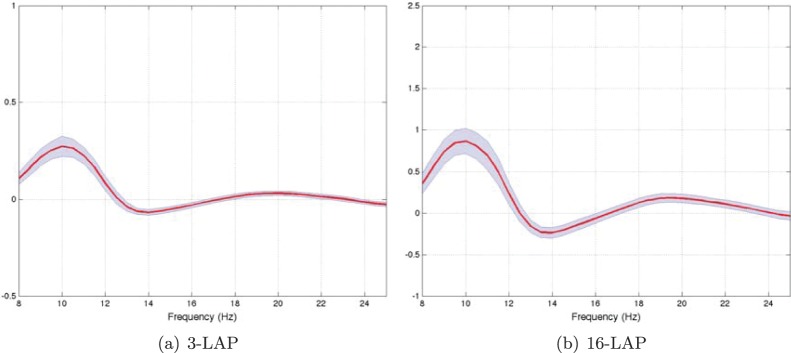
Global mean and a standard error of the resting-state EEG *pdfs* over all subjects for two channel configurations: 3 and 16 Laplacian EEG-channels. See Section 2.1 for a description of the difference between both.

A second aim of our study is to evaluate whether BSSFO is capable of predicting subjects’ BCI performance using resting-state EEG data preceding an actual BCI paradigm. Using the 1D *pdfs* of the same preprocessed resting-state EEG data (see Section 2.3), we study the dependence on the number of channels necessary for a meaningful clustering and whether the derived groups have physiologically reliable spatial and temporal features allowing for a typecasting of the subjects. Fig. 11 shows a grand mean and its standard error of the resting state *pdfs* for two different channel arrangements (3-LAP and 16-LAP).

Although the scale is different between Fig. 10 and Fig. 10, the global shapes are similar between the small and large channel arrangement. Both present a global peak around the *μ*-band and the second largest global peak around the *β*-band (in line with [20]).

**Figure 12:**
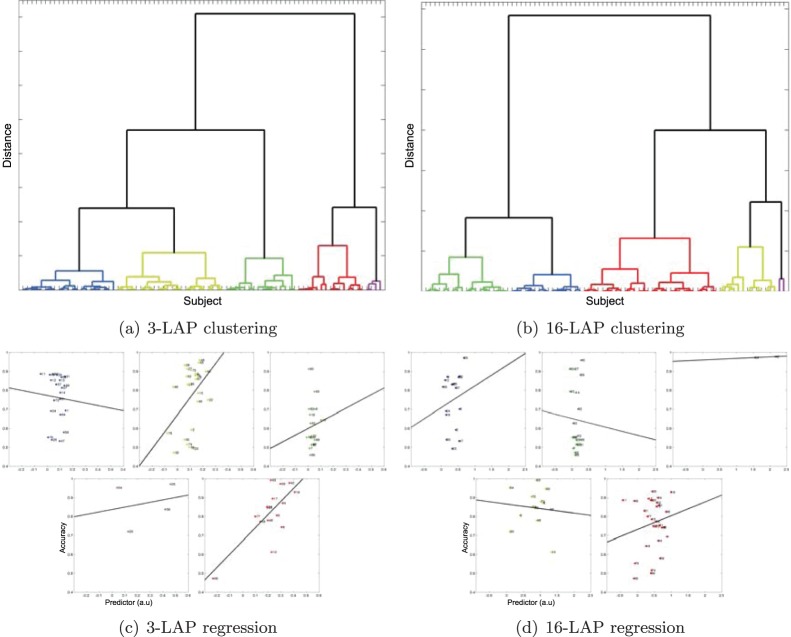
An example of a clustering result obtained by BSSFO from the resting-state EEG. (a,b) show hierarchical clustering results for a small (3) and large (16) channel configuration. For the same channel configurations, we show the corresponding cluster-wise linear regression models (c,d) between the BSSFO’s *pdfs* and the classification accuracy in the actual BCI. The BSSFO combines the cluster-wise linear regression models in performance prediction.

In Fig. 12, we illustrate the clustering results and the linear regression functions fitted to the data of each cluster. Fig. 12(a) and Fig. 12(b) show the results of the hierarchical clustering method with 3-LAP and 16-LAP channel arrangements, respectively. We selected 5 clusters for both small and large channel arrangements. The linear regression models for each cluster are given in Fig. 12(c) and Fig. 12(d).

For both channel arrangements, we can identify 2–3 high performing groups, one containing only small number of subjects. At least one cluster contains subjects with mixed performances although the predictor obtained from the resting-state EEG data has similar AUCs. Neither in the large nor the small channel arrangement a clear group of users unable of BCI communication appears. Also a small channel arrangement does not lead to significantly worse results, which is encouraging from the practical point of view.

**Figure 13:**
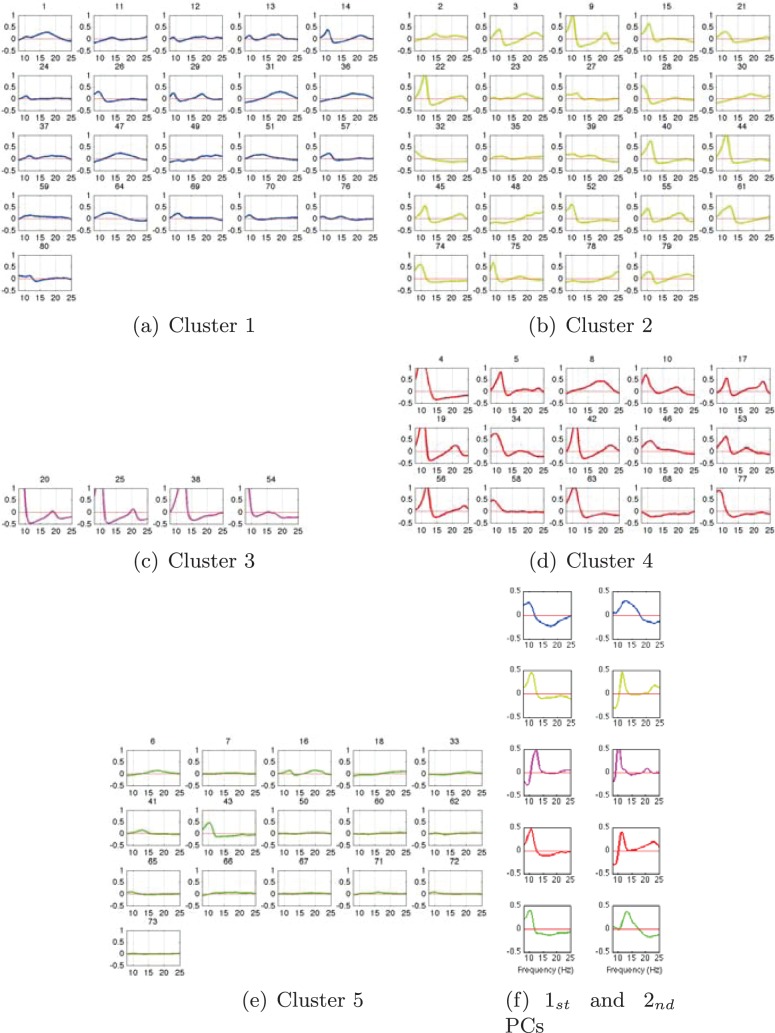
3-LAP-channel resting-state EEG *pdfs* assigned to each cluster and the corresponding two-largest Principal Components (PCs). The colors denote cluster labels from Fig. 12(a).

**Figure 14:**
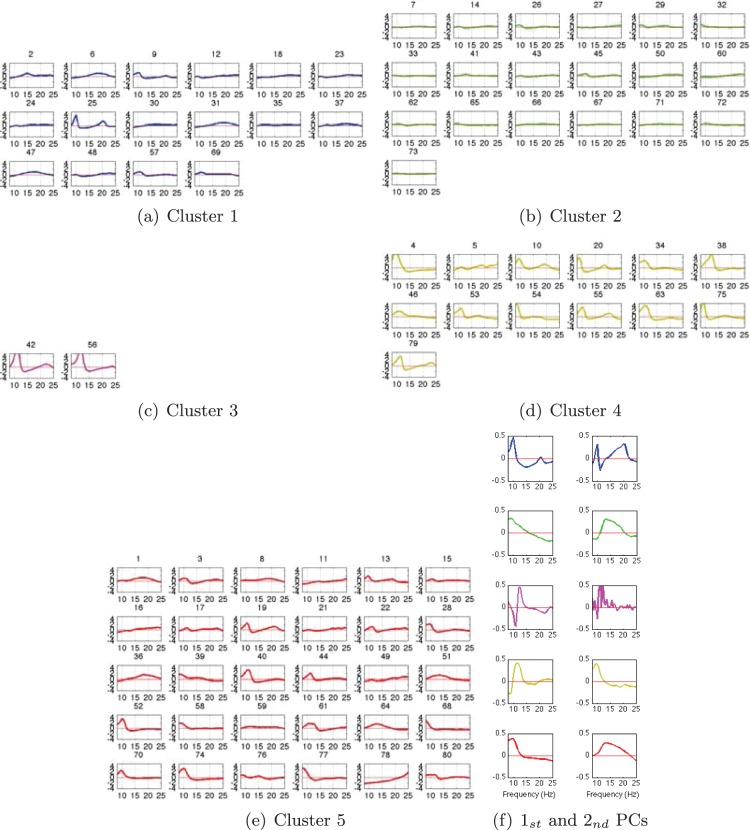
16-LAP-channel resting-state EEG *pdfs* assigned to each cluster and the corresponding two-largest Principal Components (PCs). The colors denote cluster labels from Fig. 12(b).

A close look into the cluster-wise spectral properties is given in Fig. 13 for the small channel arrangement. Here, we display each subject’s *pdf* assigned to each cluster as well as their principal components of the two largest eigenvalues. Considering the spectral features within each cluster for the 3-LAP channel arrangement, it can be stated that cluster 3 and 4 show clear peaks around the *μ*-band within each of the subject. While cluster 1 consists of subjects having either a high *μ*-, a high *β*-band or both, cluster 2 and 5 contain subjects with either no or only slight *μ*-bands. Nevertheless, the first principal component show that the *μ*-band is most prominent in all clusters, but has a specific maximum in each cluster. The second principal component shows the *β*-band again with a specific peak frequency in every cluster. For the same analysis with the large channel arrangement see Fig. 14, clearly the results are less pronounced.

**Figure 15:**
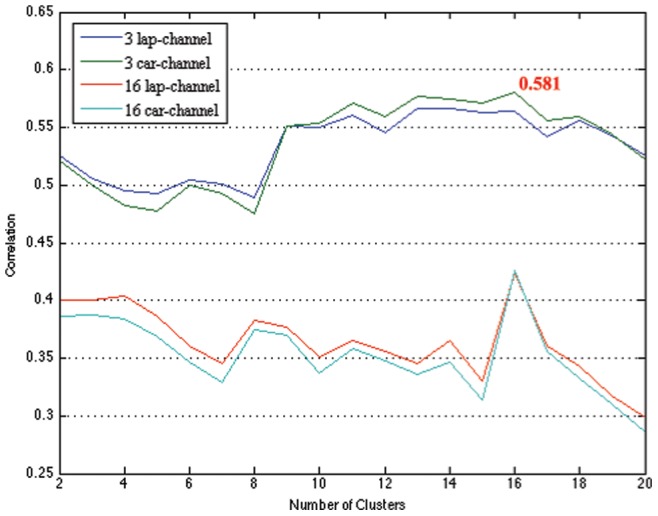
The changes of the correlation between the proposed predictor and the classification performance according to the number of clusters and the number of channels considered in prediction.

In Fig. 15, we contrast the predictors from the resting-state EEG data and the classification performance in the BCI for different numbers of channels and numbers of clusters. The maximum correlation of 0.581 for sixteen clusters was obtained. Clearly, the small number of channels positioned on the sensorimotor cortex gives rise to better correlation results when compared to the larger and more unspecific channel number that covers the whole brain.

**Figure 16:**
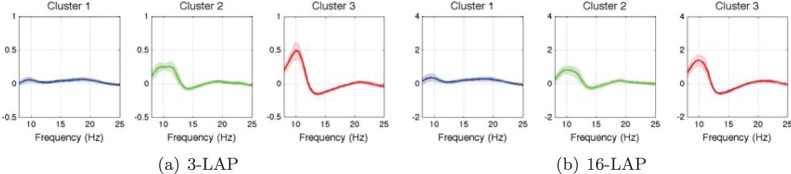
Mean and standard error of resting state EEG *pdfs* over the subjects assigned to each cluster of motor imagery *pdfs*. The cluster labels denoted with different colors are from Fig. 10.

Finally, we come back to the clustering of the *pdfs* acquired for the BCI experiment. Ideally the clusters may tell whether the resting-state *pdfs* have physiologically meaningful information especially when comparing to the *pdfs* from motor imagery. We computed the mean of the resting-state *pdfs* for each cluster trained from the motor imagery *pdfs* over the subjects. The clusters presented in Fig. 10 revealed three groups of different performance levels. While the red cluster shows the high performance group, the blue one is the worst, and the green one exhibits mediocre performance. From Fig. 16, we can clearly see that the higher the classification performance, the larger values are found in the *pdf* around the *μ*- and *β*-bands. Therefore, based on our prediction and grouping analysis, it is expected that a subject who falls into the blue group can be a potential BCI-illiteracy.

## Conclusion

In this work, we show that BSSFO evaluates favorably compared to prototypical spatio(-temporal) filtering methods like CSP [16] in terms of classification performance across a large corpus of 80 subjects from [20], and BSSFO can also infer subject-specific spatio-temporal patterns, which are shown physiologically meaningful. Individual BSSFO patterns can be clustered to form groups of subjects with similar physiological characteristics. It, therefore, may allow to gain further insight into the characteristics responsible for the performance of subjects beyond the mere amplitudes of *μ*- and *β*-bands. We could show that a clustering into three groups of subjects exhibit different spatial topographies and is highly predictive for the subjects BCI performance.

Moreover, we study the prediction of a subject’s future BCI performance based on resting-state EEG data acquired prior to a BCI session. Using only 3-Laplacian channels, we could obtain the maximum correlation coefficient of 0.581 with the performance later seen in the actual BCI feedback session; this result compares favorably with previous results [20]. A clustering of the resulting BSSFO patterns shows interesting task-independent physiological characteristics discriminative for “good” and “bad” BCI performers. It is noteworthy that unlike the earlier study [20] that assumed a statistical model of resting-state EEG, BSSFO extracts a full spectral characteristics along with the spatial properties for a subject in a data-driven manner without a-priori assuming a specific role of particular frequency bands. Therefore, it is expected that the BSSFO can be a potential tool for BCIs to care the patients who might have unusual spatio-temporal characteristics due to neurological disorder or brain injury.

Although we have performed the validation of the BSSFO framework here within an offline study, our methods may be readily applied in feedback BCI experiments, both for pre-screening subjects and for improving the spatio-temporal signal processing. The subject groupings extracted by our approach could in the future also contribute to create improved subject-independent classifiers [30], [31], [28] or better co-adaptive BCI training protocols [12].

While we focused on the SMR-controlled BCI, we would like to emphasize that the BSSFO is also applicable to other kinds of single-trial EEG signal recognition problems that are based on the modulations of brain rhythms. Therefore, it is by no means limited to SMR-controlled BCIs. Furthermore, regarding ECoG-based BCIs, which are also of great interests in the field, it has been studied that the spectral amplitudes of the ECoG signals in the various frequency bands are task-related, *e.g.*, motor movement [32], [33] or auditory processing [34]. Hence, it is natural to extend the current study to the ECoG-based BCI studies using the same framework, in which the task-related frequency bands can be effectively represented in a probabilistic manner.

## Appendix

To implement our prior knowledge of common characteristic frequency bands, we first denote 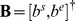 as a continuous random vector for a frequency band, where 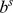 and 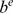 are, respectively, the start and the end frequency of this band with the constraint of 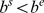. We define the probability of a frequency band **b**, *p*(**b**), as the probability that the **b** bandpass-filtered signals can be correctly classified between two classes.

Since we are presumably uncertain about the discriminative frequency band, we encode this uncertainty as a prior distribution *p*(**B**) over a random variable **B**. Given a set of single-trial EEGs 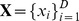 and the corresponding class labels 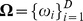, where 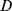 is the number of trials, we can compute the posterior *pdf*, 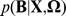, by a Bayes rule as follows:
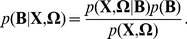
(16)


The prior, *p*(**B**), describes the relative probabilities of different states, *i.e.*, frequency bands, in which single-trial EEG responses to motor imagery are correctly discriminated. The term 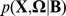 is called the *likelihood function*. If the hypothesis **B**, *i.e.*, the chosen frequency band, were true, this term indicates the probability that the single-trial EEG responses **X** are in conjunction with the class labels **Ω**. In other words, given a particular frequency band, this likelihood function describes the probability that the single-trial EEGs **X** can be correctly classified into **Ω**. The posterior distribution 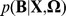 defines the probability that the frequency band **B** is discriminative when the observations of **X** and Ω are given. Thus, it indicates the relative likelihood of the single-trial EEG responses **X** being correctly classified into **Ω** by **B** bandpass filtering along with the ensuing computational processes. Note that, in this paper, we do not make any functional assumption about the densities *p*(**B**) and 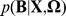, which could be linearity, Gaussianity, unimodality, etc.

Given a frequency band **B** and raw EEG signals **X**, the bandpass-filtered signals **Z** are deterministically obtained. Furthermore, a spatial filter **W** is found from **Z** via a standard CSP algorithm [12] or its variants [8], [36], [25], in which **W** is analytically obtained by optimization. In the prevalent processing chain of SMR-controlled BCIs, a feature vector is extracted by computing simple matrix multiplication between **Z** and **W** and the second-order statistics followed by a monotonically increasing logarithmic function. This means, that the posterior 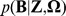 can be indirectly estimated from 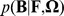, where 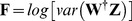, without losing information.

Hence, we can rewrite Eq. (16) as follows.
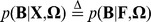





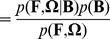
(17)where 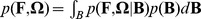. Thus, the goal of finding the optimal spatio-spectral filter to extract discriminative features and, thereby, to ultimately improve classification accuracy, can be defined as an estimation of the posterior *pdf* 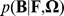 in Eq. (17) (see [24] for details and implementation).
